# AluY-mediated germline deletion, duplication and somatic stem cell reversion in *UBE2T* defines a new subtype of Fanconi anemia

**DOI:** 10.1093/hmg/ddv227

**Published:** 2015-06-17

**Authors:** Elizabeth L. Virts, Anna Jankowska, Craig Mackay, Marcel F. Glaas, Constanze Wiek, Stephanie L. Kelich, Nadine Lottmann, Felicia M. Kennedy, Christophe Marchal, Erik Lehnert, Rüdiger E. Scharf, Carlo Dufour, Marina Lanciotti, Piero Farruggia, Alessandra Santoro, Süreyya Savasan, Kathrin Scheckenbach, Jörg Schipper, Martin Wagenmann, Todd Lewis, Michael Leffak, Janice L. Farlow, Tatiana M. Foroud, Ellen Honisch, Dieter Niederacher, Sujata C. Chakraborty, Gail H. Vance, Dmitry Pruss, Kirsten M. Timms, Jerry S. Lanchbury, Arno F. Alpi, Helmut Hanenberg

**Affiliations:** 1 Department of Pediatrics and; 2 Medical and Molecular Genetics, Indiana University School of Medicine, Indianapolis, IN 46202, USA,; 3 Department of MRC Protein Phosphorylation and Ubiquitylation Unit, College of Life Sciences, University of Dundee, Dundee, UK,; 4 Department of Otorhinolaryngology and Head/Neck Surgery (ENT) and; 5 Department of Experimental and Clinical Hemostasis, Hemotherapy and Transfusion Medicine, Heinrich Heine University, Düsseldorf, Germany,; 6 Hematology Unit, G. Gaslini Children's Hospital, Genoa, Italy,; 7 Pediatric Hematology and Oncology Unit, A.R.N.A.S. Ospedale Civico, Palermo, Italy,; 8 A.O. Ospedali Riuniti Villa Sofia-Cervello, Palermo, Italy,; 9 Department of Pediatrics, Children's Hospital of Michigan, Wayne State University School of Medicine, Detroit, MI, USA,; 10 Department of Biochemistry and Molecular Biology, Boonshoft School of Medicine, Wright State University, Dayton, OH 45435, USA,; 11 Department of Gynecology, Heinrich Heine University, Düsseldorf, Germany and; 12 Myriad Genetics Inc., Salt Lake City, UT, USA

## Abstract

Fanconi anemia (FA) is a rare inherited disorder clinically characterized by congenital malformations, progressive bone marrow failure and cancer susceptibility. At the cellular level, FA is associated with hypersensitivity to DNA-crosslinking genotoxins. Eight of 17 known FA genes assemble the FA E3 ligase complex, which catalyzes monoubiquitination of FANCD2 and is essential for replicative DNA crosslink repair. Here, we identify the first FA patient with biallelic germline mutations in the ubiquitin E2 conjugase UBE2T. Both mutations were aluY-mediated: a paternal deletion and maternal duplication of exons 2–6. These loss-of-function mutations in UBE2T induced a cellular phenotype similar to biallelic defects in *early* FA genes with the absence of FANCD2 monoubiquitination. The maternal duplication produced a mutant mRNA that could encode a functional protein but was degraded by nonsense-mediated mRNA decay. In the patient's hematopoietic stem cells, the maternal allele with the duplication of exons 2–6 spontaneously reverted to a wild-type allele by monoallelic recombination at the duplicated aluY repeat, thereby preventing bone marrow failure. Analysis of germline DNA of 814 normal individuals and 850 breast cancer patients for deletion or duplication of *UBE2T* exons 2–6 identified the deletion in only two controls, suggesting aluY-mediated recombinations within the *UBE2T* locus are rare and not associated with an increased breast cancer risk. Finally, a loss-of-function germline mutation in *UBE2T* was detected in a high-risk breast cancer patient with wild-type *BRCA1/2*. Cumulatively, we identified *UBE2T* as a bona fide FA gene (*FANCT*) that also may be a rare cancer susceptibility gene.

## Introduction

Fanconi anemia (FA) is an autosomal or X-linked recessive inherited DNA instability disorder that is caused by germline mutations in at least 17 genes (*FANCA, FANCB, FANCC, FANCD1/BRCA2, FANCD2, FANCE, FANCF, FANCG, FANCI, FANCJ/BRIP1, FANCL, FANCM, FANCN/PALB2, FANCO/RAD51C, FANCP/SLX4, FANCQ/ERCC4* and *FANCS/BRCA1*) that are involved in the DNA damage response (1–4). Clinically, FA is characterized by variable congenital abnormalities, progressive bone marrow failure, endocrine abnormalities and a predisposition to malignancies, primarily acute myelogenous leukemia and solid tumors (5–8). In the absence of congenital abnormalities, FA is usually diagnosed based on the onset of bone marrow failure ranging from mild to severe within the first decade of life (5,9,10). An analysis of 754 North American FA patients enrolled in the International Fanconi Anemia Registry (IFAR) demonstrated that the average age of hematological onset of FA is 7.6 years (5). However, as *FANCA*, *FANCC* and *FANCG* are the most frequently mutated FA genes (5,9,11), the onset of bone marrow failure might differ in FA patients with rarer gene defects (2,4,12–15). If bone marrow failure does not occur due to the presence of a ‘milder’ mutation with residual protein function or due to mosaicism in the hematopoietic system as a consequence of a gain-of-function mutation in hematopoietic stem cells (16–19), the diagnosis of FA is often made upon presentation with cancer or with severe toxicity after treatment of a malignancy with chemotherapy (5,20,21).

Cells from FA patients exhibit a distinctive cellular phenotype of hypersensitivity to DNA interstrand crosslinking agents such as mitomycin C (MMC) and diepoxybutane (DEB), which can be assessed as increased chromosomal breakage in metaphase and by G2 cell cycle arrest using flow cytometry (22–24). Upon recognition of a stalled replication fork in S phase at a DNA interstrand crosslink (ICL), a core protein complex formed by the products of eight FA genes (*A/B/C/E/F/G/L/M*) is activated via ATR-mediated phosphorylation and recruited to the sites of ICL lesions. Subsequently, FANCL, the FA core complex subunit with E3 RING ligase activity, monoubiquitylates the FANCI/FANCD2 (I/D2) protein dimer formed by the products of the two FA genes *FANCD2* and *FANCI* at corresponding lysine residues in each protein (3,25,26). This activation process is critically dependent on the presence of all eight FA core complex gene products and additional accessory FA-associated proteins such as FAAP20, −24 and −100. The E2 conjugase UBE2T, which is thought to be recruited independently of the FA core complex to damaged chromatin, specifically binds to FANCL to promote the site-specific monoubiquitination of FANCD2 and FANCI (27–32). The products of the other FA genes *D1/BRCA2*, *J/BRIP1*, *N/PALB2*, *O/RAD51C*, *P/SLX4*, *Q/ERCC4*, and *S/BRCA1* are dispensable for the monoubiquitination of FANCD2 and FANCI and therefore are classified as *downstream* components of the FA pathway (3,4,25,26). These FA proteins are involved in later stages of the ICL repair and are essential players in homologous recombination (HR) repair. Importantly, heterozygous germline mutations in these late/downstream genes predispose individuals to several malignancies, such as breast, ovarian and pancreatic cancers (33–35).

## Results

### Clinical presentation

The most distinct and indicative cellular defects in FA patient-derived cells are hypersensitivity to low doses of DNA crosslinking agents, such MMC or DEB, and high frequencies of chromosomal abnormalities (36). Even today, a small percentage of individuals are diagnosed by pathological chromosomal breakage tests, but exhibit no pathogenic mutations in known FA/DNA repair genes. One such individual is the 16-year-old FA patient 100166/1 who has American parents with solely Italian ancestry. Except for the thalassemia trait, this patient has no family history of genetic predisposition for cancer or an increased miscarriage frequency. The patient was born with bilateral malformations of both thumbs and radii and small stature. Within the first week of life, he was diagnosed as being affected by FA due to high levels of DEB-induced high chromosomal breakage in metaphases of hematopoietic cells: baseline 0.1 and 5.8 breaks/cell in the absence or presence of 0.1 µg/ml DEB (normal 0.0–0.05 and 0.00–0.1, respectively). However, a facial appearance atypical for FA, normal bone marrow cellularity, normal leukocyte and thrombocyte counts after the perinatal period, mild anemia with a low mean corpuscular volume (Supplementary Material, Fig. S1) due to a thalassaemia minor mutation inherited from his father, and the failure to identify germline mutations in DNA repair genes prevented a clear genetic diagnosis for the first 16 years of his life.

### A defect in FANCD2 monoubiquitination in the patient's fibroblasts

**Figure 1.**
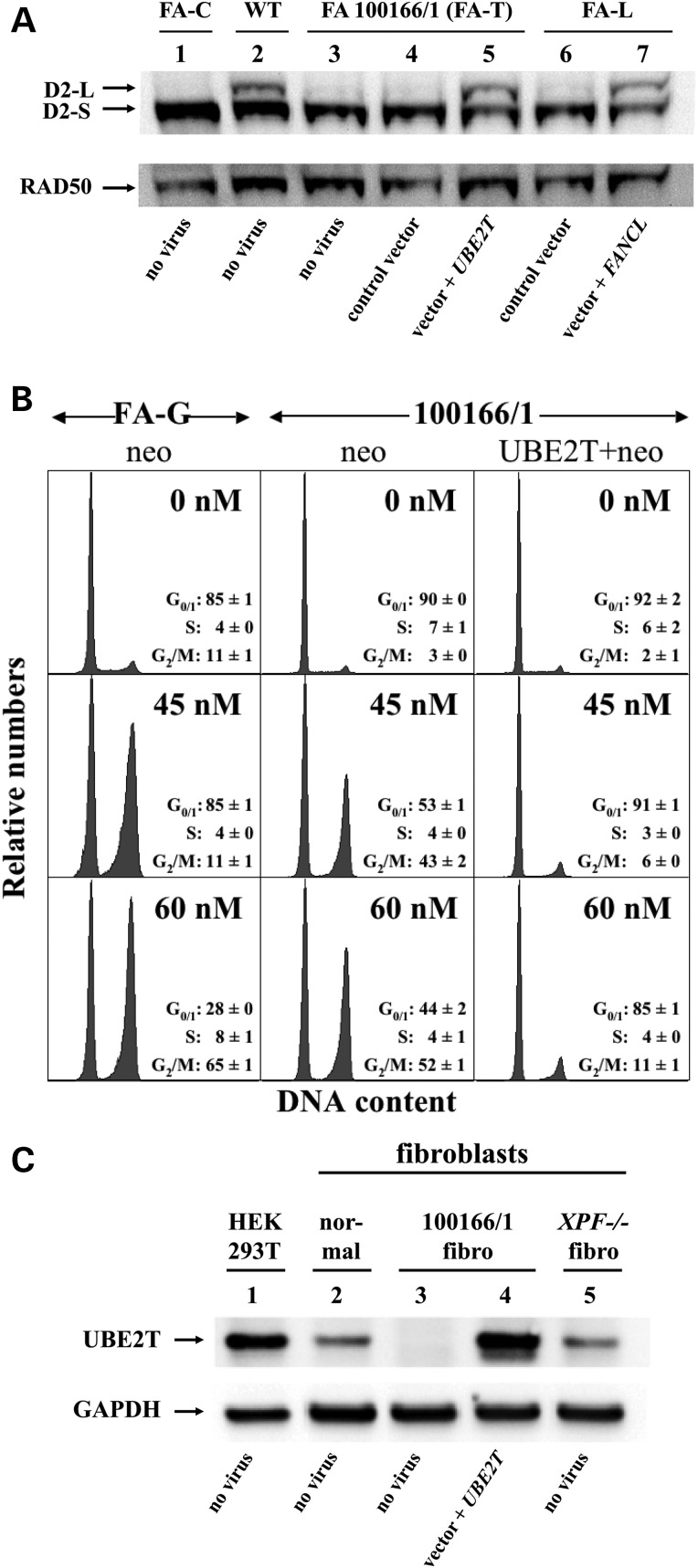
Identification of *UBE2T* as new FA gene by retroviral complementation of FA 100166/1 fibroblasts and western blotting. (**A**) Western blot analysis of protein extracts from transduced fibroblast lines immunostained with a FANCD2 antibody to detect non-ubiquitinated (D2-S) and monoubiquitinated (D2-L) FANCD2. Lanes 1–3 depict the FANCD2 ubiquitination status of *FANCC*-deficient (FA-C), wild-type (WT) and FA100166/1 fibroblasts exposed to 150 nm MMC overnight. Lanes 4–7 show the FANCD2 monoubiquitination status of G418-resistant FA 100166/1 and FANCL-deficient fibroblasts, genetically modified with a control vector or with vectors expressing *UBE2T* or *FANCL* cDNAs, respectively. Staining with a RAD50 antibody was used to visually confirm equal loading. (**B**) Flow cytometric cell cycle analysis of transduced primary fibroblasts grown for 3 days in 0, 45 or 60 nm MMC. FA-G: primary *FANCG−/−* fibroblasts (38) transduced with the control vector (neo control). FA 100166/1: primary patient fibroblasts transduced with the control vector (neo control) or the UBE2T-coexpressing vector (UBE2T + neo). The distribution of cells in G_0_/G_1_, S and G_2_/M arrest from three different experiments is shown. (**C**) Western blot analysis of protein extracts from the indicated cells upon staining with a polyclonal antibody specific for human UBE2T protein. HEK293T cells, immortalized fibroblasts from a healthy donor (normal) and from a *FANCQ/ERCC4/XPF* deficient (*XPF−/−*) patient were used as positive controls. Analysis of non-transduced or transduced immortalized FA patient cells (FA 100166/1) showed specific restoration of the absent UBE2T protein by the UBE2T retrovirus. Staining with a GAPDH antibody was used to visually confirm equal loading.

For initial diagnostic classification, we performed a standard chromosomal breakage analysis using DEB as a genotoxin in the primary FA 100166/1 skin fibroblasts obtained from the patient at 2 years of age. The results shown in Table 1 clearly demonstrate that the fibroblast cells possessed the typical characteristic hypersensitivity of FA cells towards DEB, compared with normal fibroblasts and the highly sensitive fetal *FANCG−/−* reference fibroblasts. As an established diagnostic tool in the classification of FA patients (37), we next performed western blotting analysis on immortalized FA100166/1 fibroblasts, which revealed that exposure of the cells to MMC overnight did not lead to monoubiquitination of FANCD2 (Fig. 1A, lane 3), a central activation step in the FA pathway that is dependent on the normal function of the ‘early’ FA genes and also includes *FANCI* (1). Therefore, we transduced primary skin fibroblasts of the patient with a series of G418-selectable retroviral vectors that expressed the cDNAs of *FANCA/B/C/E/F/G/L/I* similarly, as described previously (13). Stably transduced G418-resistant primary fibroblasts were incubated with low doses of MMC (0, 45 or 60 nm). Cell cycle analysis after 3 days revealed that the patient's fibroblasts were clearly hypersensitive to the ICL lesions. However, none of the retroviral vectors corrected the MMC-induced G2 arrest of the fibroblasts (Supplementary Material, Fig. S2). We therefore hypothesized that this patient had a defect in a yet unidentified FA gene.

**Table 1.** Metaphase chromosomal breakage analysis of primary fibroblasts

Sample	DEB dose (µg/ml)	Number of metaphases scored	Total number of breaks	Breaks per Cell
Fetal fibroblast 94–38	0	66	4	0.06
(*FANCG−/−*)	0.01	49	47	0.96
	0.1	1	5	5.00
FA 100166/1	0	52	15	0.29
(*UBE2T−/−*)	0.01	50	44	0.88
	0.05	50	101	2.02
	0.1	50	140	2.80
NL1433889	0	50	0	0.00
(normal control)	0.01	47	2	0.04
	0.1	47	7	0.15

### Retroviral complementation identifies *UBE2T* as a new FA gene

Candidate genes (*FAAP20, FAAP24, FAAP100* and *UBE2T*), for which deficiencies have been associated with defective FANCD2 monoubiquitination (27,39–43), were introduced into FA 100166/1 primary skin fibroblasts using G418-selectable retroviral expression vectors (13). Of all analyzed cDNAs, only expression of *UBE2T* (RefSeq: NM_014176.3) completely abrogated the cellular hypersensitivity towards 45 and 60 nm MMC (Fig. 1B and Supplementary Material, Fig. S3). Western blot analysis of whole cell lysates revealed that FANCD2 monoubiquitination was restored in *UBE2T*-transduced cells comparable to the FANCD2-L levels (D2-L) obtained in wild-type (WT) fibroblasts and in FANCL-deficient fibroblasts complemented by a specific *FANCL* virus (Fig. 1A lanes 2, 5 and 7). Using a polyclonal UBE2T antibody (44), we demonstrated that the endogenous UBE2T protein was largely absent in non-transduced and control vector-transduced patient fibroblasts, but abundantly detected in cells transduced with the UBE2T-expressing vector (Fig. 1C lanes 3 and 4). We and others previously showed that FANCL also binds to another E2 conjugase, UBE2W (29,45). However, expression of UBE2W in fibroblasts from patient 100166/1 did not rescue the MMC-induced cell cycle arrest (Supplementary Material, Fig. S4). These results strongly suggest that the patient inherited two germline mutations in *UBE2T* and that this E2 conjugase is the 18th FA gene, *FANCT*.

### Identification of two germline mutations, a deletion and a duplication, in *UBE2T* due to aluY-mediated recombination

To confirm the results of the functional complementation and biochemical experiments, each exon of the *UBE2T* gene including the splice junctions was sequenced using the genomic DNA of patient 100166/1 fibroblasts. We could not identify any pathogenic mutation in the genomic DNA at the *UBE2T* locus either by capillary Sanger or whole exome sequencing (data not shown). We also did not detect any pathogenic mutations in the genomic 5′ promoter and the 3′ untranslated regions (UTRs) of the *UBE2T* gene (data not shown). Sanger sequencing confirmed the presence of two 311-bp aluY elements in identical orientation in intron 1 and intron 6 with 5′ long stretches of T nucleotides (Supplementary Material, Fig. S5). In our patient, both aluY elements were 100% identical, in contrast to the current assembly of the human genome, GRCh38.p2, where the two aluY elements contain a single mismatch at position 192. Surprisingly, sequencing of a large proportion of the introns in genomic DNA from the FA 100166/1 fibroblasts revealed the presence of only three heterozygous single nucleotide polymorphisms (SNPs), one of which was located in intron 6 (Supplementary Material, Fig. S6), thus suggesting a large genomic deletion in at least one allele. However, semi-quantitative PCR for all seven exons of *UBE2T* suggested that all exons were present in the fibroblasts at normal dosages (data not shown). In addition, linkage analysis performed on the family using SNPs on the Affymetrix 6.0 array did not indicate that the parents were consanguineous for the *UBE2T* locus on chromosome 1 (data not shown).

**Figure 2.**
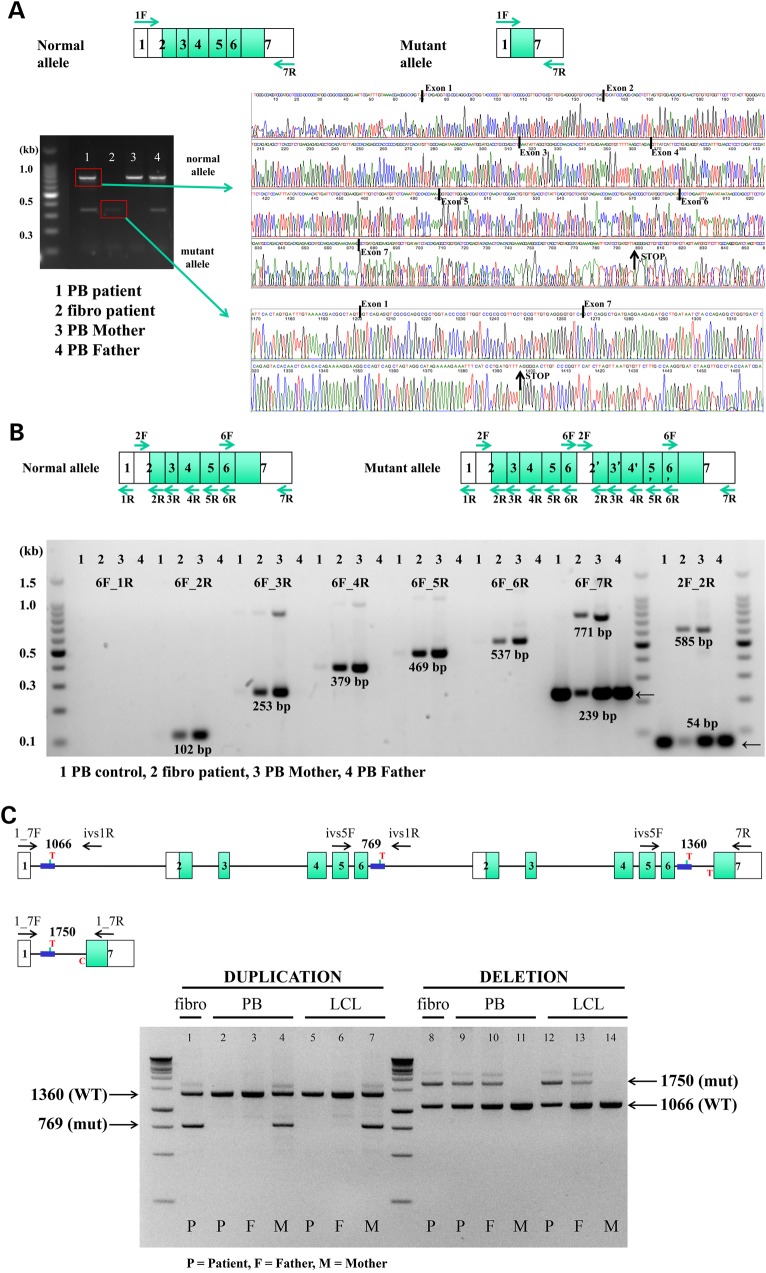
Identification of two aluY-mediated germline mutations in *UBE2T* in FA 100166/1. (**A**) Identification of the paternal exons 2–6 deletion in *UBE2T* in cDNA. Forward and reverse primers in exons 1 (1F) and 7 (7R) were utilized in cDNA from the peripheral blood (PB) and fibroblasts (fibro) of the FA 100166/1 patient. The PB from the patient was sampled at 14 years of age. cDNA was also obtained from the PB of the mother (PB Mother) and father (PB Father). The amplified PCR products were run on a gel, and the bands excised and sequenced. Sequencing traces for two of the band are shown. Boundaries of the different exons are indicated. (**B**) Identification of the maternal exons 2–6 duplication in *UBE2T* in cDNA. cDNA was generated from the primary fibroblasts of the patient (Fibro patient) and the PB of a normal individual (PB control), the mother (PB Mother) and the father (PB Father) and subjected to PCR. The locations of the primers are shown on the diagrams, and the primer combinations are indicated on the gel. The sizes for the amplified PCR products of UBE2T are indicated. All bands were excised and sequenced. (**C**) Detection of the deletion and duplication of exons 2–6 in genomic DNA of family members. Two distinct PCRs were developed using primers for co-amplification of the normal as well as a mutant allele in a single reaction. The locations of the primers and the expected sizes of the PCR products are shown in the top diagram. Genomic DNA from the patient's (P), the Father's (F) and the Mother's (M) peripheral blood (P) and EBV-transformed LCLs (LCL) was analyzed. In addition DNA from the patient's fibroblasts (fibro) was used. The sizes and identities of the PCR products are shown on the left and right sides of the gel. Mutant bands were excised and sequenced.

To test for a deletion, we used exon 1F and 7R primers in cDNA and amplified a normal allele from the sample obtained from the patient at 14 years of age, as well as samples from the mother and father (Fig. 2A, lanes 1, 3 and 4). We detected a shorter mutant allele in the cDNA from the patient's peripheral blood and fibroblasts and in that from the father (Fig. 2A, lanes 1, 2 and 4). Sequencing of the PCR products confirmed the presence of the normal allele and revealed that the mutant *UBE2T* allele inherited from the father was missing exons 2–6 (Fig. 2A).

We hypothesized that a more complex genomic rearrangement might have occurred on the maternal allele. To test for a duplication within the *UBE2T* locus, we generated cDNA using the primary fibroblasts from the patient and peripheral blood from a normal individual and from the mother and father. Surprisingly, we were able to specifically amplify a 585-bp product with a pair of non-overlapping exon 2F and exon 2R primers (Fig. 2B 2F_2R, lanes 2 and 3) that generated a 54-bp product of exon 2 in the normal *UBE2T* gene locus (2F_2R, lanes 1–4), thus clearly demonstrating that additional genomic material was present at the mRNA level in the patient's fibroblasts and the mother's leukocytes. Using combinations of exon 6F and exons 2, 3, 4, 5, 6 and 7 reverse primers shown in Figure 2B followed by sequencing of the amplified products revealed the presence of a duplication of exons 2–6 at the mRNA level in the patient's fibroblasts and the mother's peripheral blood (Fig. 2B, lanes 2 and 3).

In summary, we hypothesized that the patient inherited a large intragenic deletion in *UBE2T* from his father, creating a new intron between exons 1 and 7 that contained only one aluY element and the adjacent nucleotides of the 5′ part of intron 1 and the 3′ part of intron 6, respectively (Fig. 2C). As the translational start of *UBE2T* is in exon 2, no mutant UBE2T protein is expressed from the paternal allele. From his mother, the patient inherited a duplication of exons 2–6 including all intronic sequences and one additional alu element, thus generating a *UBE2T* allele with three identical aluY elements in identical orientation (Fig. 2C).

### Reversion of the mutant allele inherited from the mother by monoallelic aluY-mediated recombination

To confirm these rearrangements in genomic DNA and to understand the appearance of a normal allele in the peripheral blood cells of the patient (Fig. 2A, lane 1), we developed two distinct PCRs as outlined in Figure 2C for co-amplifying the normal as well as a mutant allele in one reaction. The PCR analyses on genomic DNA showed that the patient inherited the aluY-mediated duplication of *UBE2T* from his mother (Fig. 2C, lanes 1, 4 and 7). However, the duplication was not present in the genomic DNA from the patient's peripheral blood nor in a EBV-transformed B-cell line newly established when the patient was 14 years of age (lanes 2 and 5). It also was not detected in the father (lanes 3 and 6). Combined, these results demonstrate that the duplication of exons 2–6 in *UBE2T* was lost in the hematopoietic system of the patient, thus proving that the normal thrombocytes and platelet counts observed after the perinatal period were due to somatic mosaicism. The strong *in vivo* selective advantage of phenotypically normal stem cells and their progeny led to replacement of the defective FA hematopoiesis over time, thus preventing the development of bone marrow failure. The second mutation inherited from the father was not subject to reversion and thus was detected in all genomic DNA from the patient and his father (Fig. 2C, lanes 8–10, 12–13), but not in the DNA from his mother (lanes 11 and 14). Sequencing of the PCR products confirmed that the rearrangements of the duplication and the deletion as well as the reversion had occurred at the aluY repeats within the UBE2T locus.

### The maternal allele encodes an mRNA for a shorter functional UBE2T protein that is degraded by nonsense-mediated mRNA decay

**Figure 3.**
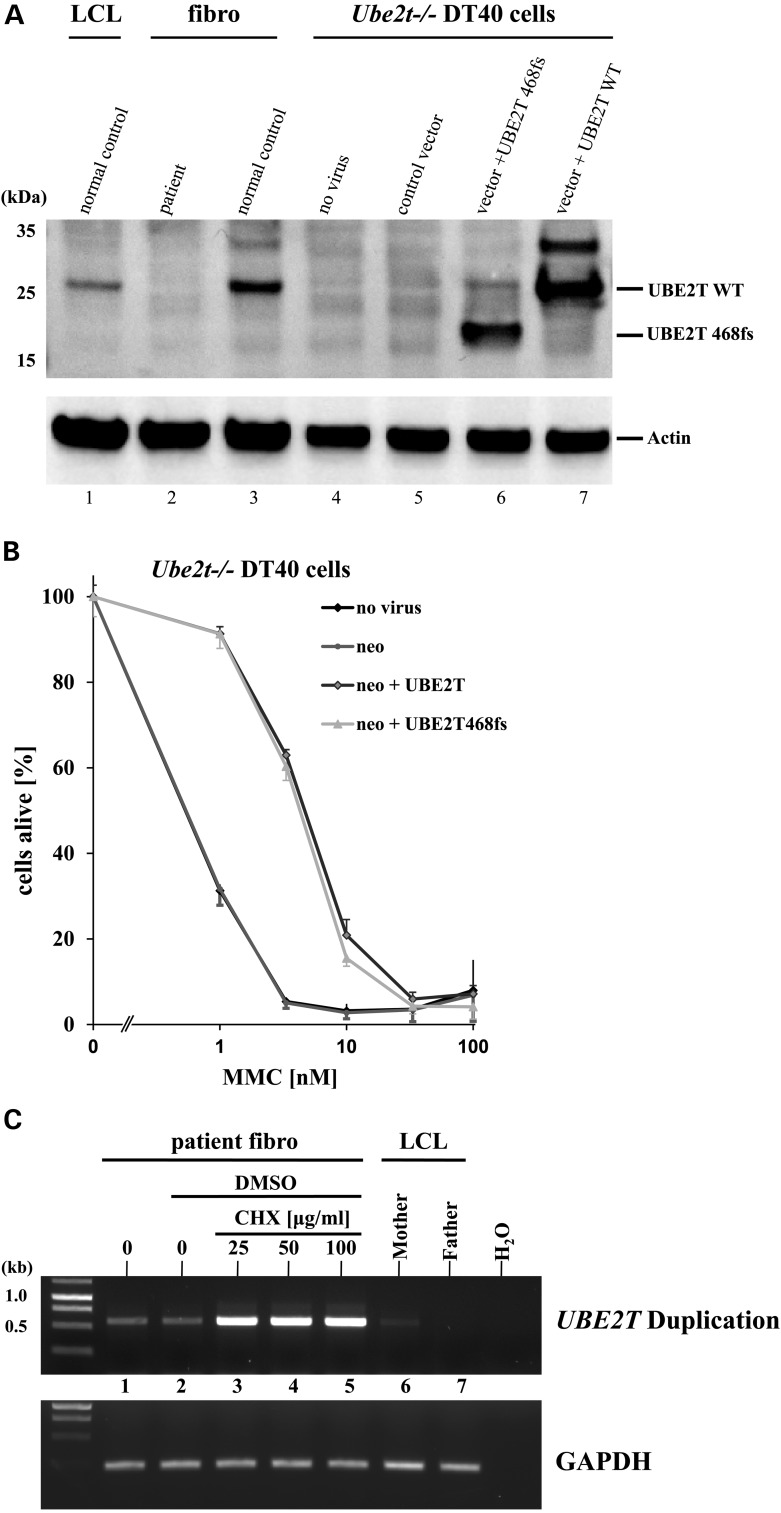
Functional analysis of the mutations and NMD. (**A**) Expression of the mutant UBE2T 468fs protein in *Ube2t−/−* DT40 cells. Cell lysates from a normal B-cell line (LCL), immortalized patient and normal control fibroblasts, non-transduced DT40 cells (no virus), and DT40 cells transduced with the control, UBE2T 468fs or WT UBE2T vectors were probed with UBE2T antibody. An antibody specific for actin was used to visually confirm equal loading. (**B**) Retroviral complementation of *Ube2t−/−* DT40 cells with the mutant UBE2T 468fs protein. Stable expression of the UBE2T WT (black, non-filled diamond) and 468fs (grey, triangle) proteins in *Ube2t−/−* DT40 cells improved survival of the DT40 cells cultured for 3 days in increasing concentrations of MMC, compared with non-transduced (black, filled diamond) and control vector (dark grey, dot)-transduced cells. (**C**) mRNA generated from the maternal allele with the duplication is subject to NMD. cDNA was obtained from immortalized FA 100166/1 fibroblasts that were incubated for 6 h in cycloheximide (CHX) as indicated. In addition, cDNA was obtained from patient's fibroblasts not cultured with DMSO (Lane 1) and from non-treated maternal or paternal EBV-transformed B-cells (LCL). PCR was performed with primers specific for the maternal duplication generating a PCR product of 566 bp. As a control, *GAPDH* cDNA was amplified from the cDNAs with specific primers under the same conditions.

Theoretically, the duplication of exons 2–6 within the maternal allele should lead to an mRNA with a shortened open reading frame encoding 162 amino acids with a frameshift at position c.469 due to the addition of the first 10 base pairs of exon 2–6 (Fig. 2C and Supplementary Material, Fig. S7). This mutant protein (UBE2T 468fs) contained the complete UBC domain (5–152) followed by six mutant amino acids (157–162), but lacked the terminal 41 amino acids of the WT protein. The first open reading frame of this mutant was followed by the correct arrangement of exons 2–7, which could encode the entire wild-type protein. We therefore cloned the first shorter mutant ORF (*UBE2T 468fs*) into a retroviral vector and stably expressed the mutant protein in *Ube2t−/−* DT40 cells. Western blotting on transduced DT40 cells demonstrated that the shorter protein was expressed at high levels (Fig. 3A). Importantly, this mutant protein was capable of ensuring the survival of *Ube2t−/−* DT40 cells when challenged with MMC, similar to WT UBE2T protein (Fig. 3B).

However, as the shorter protein of 162 amino acids from the maternal allele was never detected in the patient's fibroblasts by western blotting, we hypothesized that the mRNA generated from the maternal allele may not be stable due to the entire mRNA structure with the premature stop in the duplicated second exon 2. In general, mutations that generate premature stops can result in low levels of mutant mRNA owing to degradation by nonsense-mediated mRNA decay (NMD) (46–48), which is one of the main RNA surveillance mechanisms in cells. To test the hypothesis that the mutated *UBE2T* mRNA from the duplication was being destroyed by NMD, thereby explaining the absence of the expected UBE2T 468fs protein in the FA 100166/1 fibroblasts, we treated the fibroblasts with different doses of cycloheximide, a known inhibitor of NMD (49), and analyzed the mRNA by RT-PCR. PCR amplification revealed a clear increase of the mutant maternal allele in the fibroblast cDNA following cycloheximide treatment (Fig. 3C), demonstrating that the mRNA from the maternal duplication was indeed degraded by NMD.

### Characterization of an *early* FA phenotype in patient *UBE2T−/−* fibroblasts

**Figure 4.**
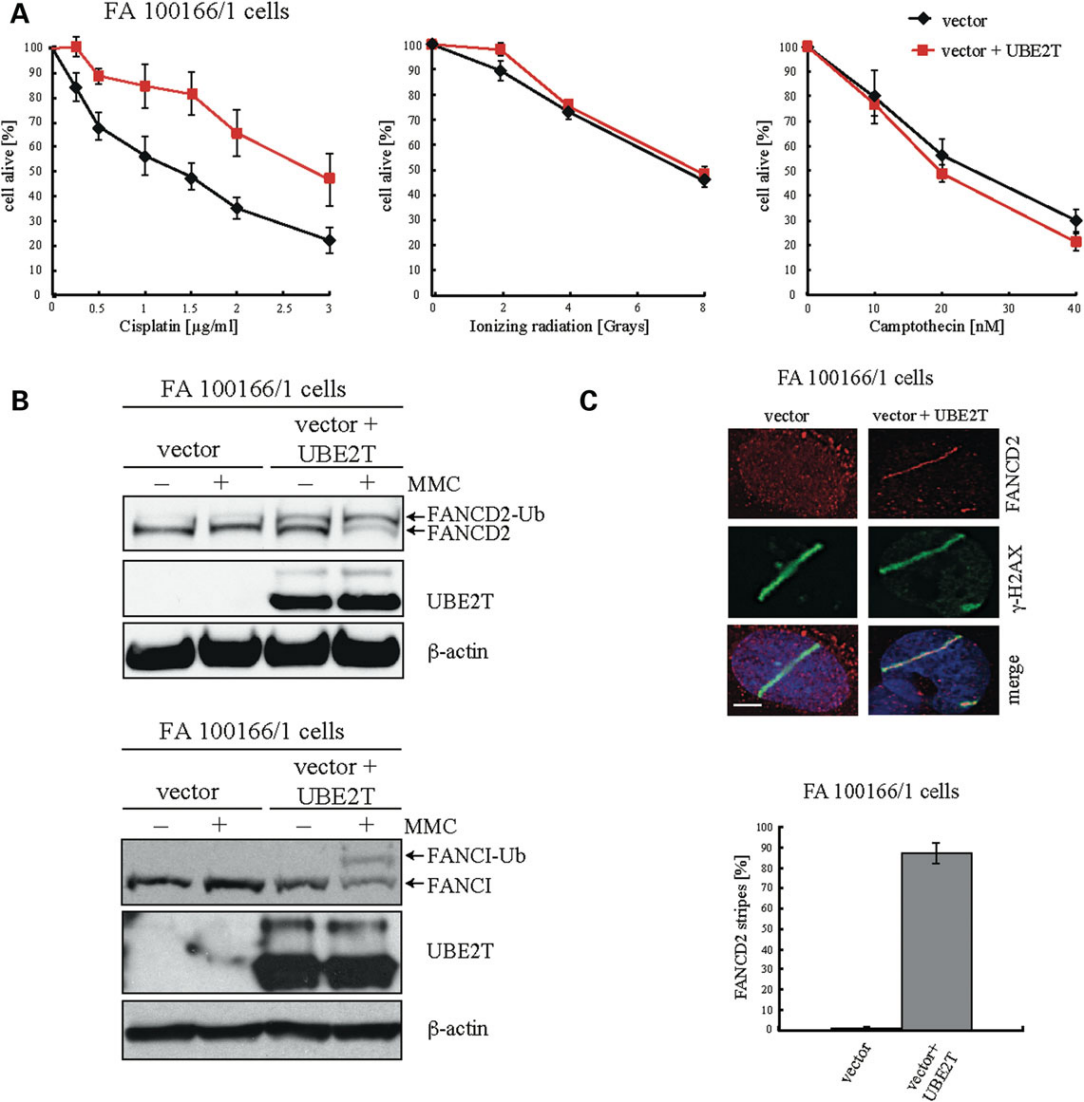
The cellular phenotype of human *UBE2T−/−* fibroblasts is comparable to that of FA cells with defects in FA core complex members. (**A**) Survival of *UBE2T−/−* fibroblasts after exposure to genotoxins. An isogenic pair of *UBE2T−/−* fibroblasts transduced with a retroviral vector encoding UBE2T or the corresponding control vector were exposed to increasing doses of the genotoxins cisplatin, ionizing radiation and camptothecin. (**B**) Western blot analysis to visualize intact FANCD2 and FANCI monoubiquitination in immortalized *UBE2T−/−* fibroblasts after exposure to MMC by UBE2T expression. (**C**) Restoration of FANCD2 recruitment to sites of DNA crosslinks by UBE2T expression. Psoralen interstrand crosslinks were induced along the track of a UVA laser in corrected and non-corrected *UBE2T−/−* fibroblasts and then visualized by γ-H2AX staining. FANCD2 accumulation at the sites of psoralen laser tracks only occurred in FA100166/1 cells that had been transduced with the UBE2T expressing retroviral vector.

We next analyzed the patient's fibroblasts for defects related to the function(s) of FA proteins. For this purpose, we used immortalized *UBE2T−/−* fibroblasts (FA 100166/1T) to generate isogenic pairs by transduction either with a UBE2T-expressing vector or the corresponding control vector and characterized their sensitivities toward known genotoxic agents (Fig. 4A). Non-corrected *UBE2T−/−* cells (control) were hypersensitive to cisplatin, but this was attenuated by UBE2T expression. In contrast, UBE2T-dependent hypersensitivity towards ionizing radiation or the topoisomerase I inhibitor camptothecin was not detected. This sensitivity profile fits well with previously published results in *Ube2t−/−* DT40 cells (44) and with other patient-derived FA cells with deficient monoubiquitination of FANCD2 (3,25,26).

We next assessed FANCD2 and FANCI monoubiquitination in response to MMC and also FANCD2 recruitment to sites of DNA lesions in the *UBE2T−/−* fibroblasts. MMC-induced FANCD2 and also FANCI monoubiquitination was not detectable in immortalized *UBE2T−/−* FA 100166/1 fibroblasts (Fig. 4B). In addition, FANCD2 recruitment to sites of interstrand crosslinks (ICLs), visualized by γ-H2AX staining of UVA laser track-induced psoralen crosslinks, did not occur in *UBE2T−/−* cells (Fig. 4C). Importantly, *UBE2T−/−* cells transduced with the UBE2T expression vector regained FANCD2 and FANCI monoubiquitination and also FANCD2 accumulation at the sites of psoralen laser tracks (Fig. 4B and C), thus confirming the essential role of the human E2 conjugating enzyme UBE2T for FANCD2 and FANCI monoubiquitination and ICL repair.

### Frequencies of aluY-mediated rearrangements in the *UBE2T* locus

**Figure 5.**
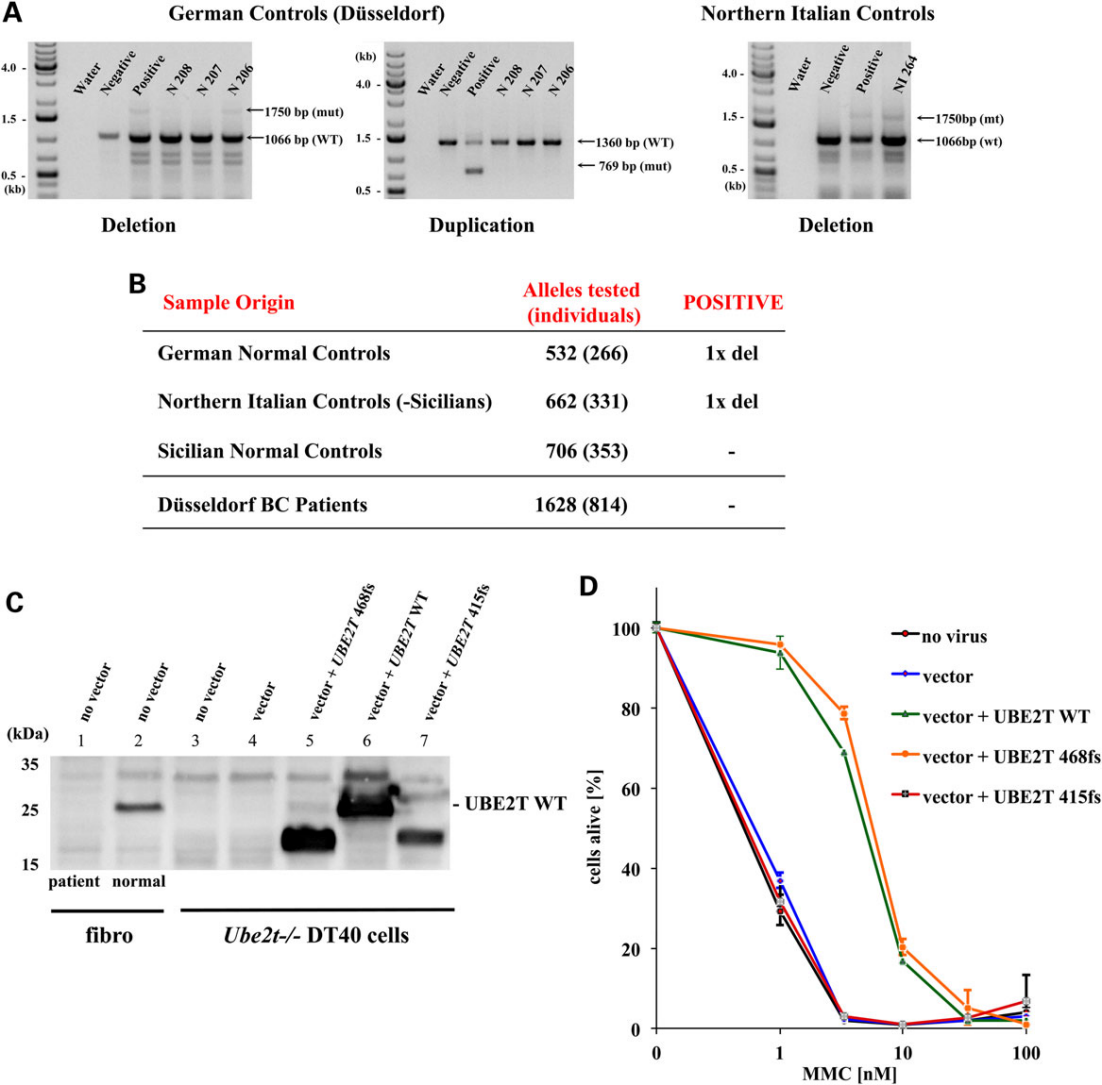
*UBE2T* mutations in normal individuals from Germany and Italy and in BRCA1/2 WT breast cancer patients. (**A**) Detection of the *UBE2T* exons 2–6 deletion in two healthy individuals. PCR analysis with primers for coamplification of the normal and mutant alleles with the exons 2–6 deletion were used to identify the deletion in genomic DNA of two normal controls, N 206 from Germany and NI 264 from Northern Italy. (**B**) Frequencies of the UBE2T exons 2–6 deletion and duplication in normal individuals and German breast cancer patients. The PCRs with primers for co-amplification of the normal and two mutant alleles with the exons 2–6 deletion and duplication were performed on genomic DNA from 850 normal control individuals from Germany and Italy. PCR analysis was also performed on genomic DNA of 814 breast cancer patients from the University of Düsseldorf breast cancer clinic. (**C**) The mutant UBE2T 415fs protein is stably expressed in *Ube2t−/−* DT40 cells. Cell lysates from the FA 100166/1 patient fibroblasts and normal control fibroblasts, non-transduced *Ube2t−/−* DT40 cells (no vector) and *Ube2t−/−* DT40 cells transduced with the control vector (vector), the WT UBE2T (UBE2T WT), the frameshift mutant UBE2T 468fs, or the frameshift mutant UBE2T 415fs vectors were immunoblotted and probed with the polyclonal UBE2T antibody. (**D**) Expression of the mutant UBE2T 415fs protein does not correct the MMC hypersensitivity of *Ube2t−/−* DT40 cells. Expression of the human UBE2T WT (blue line) and 468fs protein (green) increased the survival rates of *Ube2t−/−* DT40 chicken cells during culture with increasing concentrations of MMC for 3 days. The UBE2T 415fs mutant protein (red) did not confer any survival advantage relative to that of non-transduced (no virus, black line) and control vector-transduced (vector, blue) cells.

As both parents were of Italian origin with ancestors coming from Sicily, we next analyzed the frequencies of the duplication and deletion of *UBE2T* exons 2–6 in germline DNA from normal individuals from Northern Italy and Sicily. A total of 706 alleles from Sicily and 662 alleles from Northern Italy were tested. In addition, we analyzed the genomic DNA of 532 alleles from blood donors of German descent. Using the PCR approach described above, we detected the aluY-mediated deletion of *UBE2T* exons 2–6 in only two healthy individuals, one from Northern Italy and one from Germany (Fig. 5A and B). We did not detect the duplication in any of the 1900 alleles from the healthy controls. Unfortunately, genomic DNA from the maternal pedigree was not available for further analysis.

### Germline mutations in *UBE2T* in breast cancer patients

At least 5 of the 17 known FA genes, *D1/BRCA2*, *J/BRIP1*, *N/PALB2*, *O/RAD51C* and *S/BRCA1*, are well-established breast and/or ovarian cancer susceptibility genes (33,50,51). Although heterozygote germline mutation carriers with defects in these *late* FA genes are at an increased risk for cancers with loss/inactivation of the WT allele in malignant cells, defects in *early* FA genes have only been weakly associated with cancer (33,52). We therefore investigated the frequencies of aluY-mediated deletion and duplication in *UBE2T* in germline DNA from 814 German patients from the Düsseldorf Breast Cancer Center using the PCR-based genomic detection strategy described above. Surprisingly, we did not find any patient with either deletion or duplication of exons 2–6 in *UBE2T*, thus demonstrating that the frequency of aluY-mediated genomic rearrangements in the *UBE2T* locus was not increased in this cancer patient population (Fig. 5B).

To ultimately define the role of *UBE2T* germline mutations in patients with breast/ovarian cancer, we performed whole exome sequencing using the Ilumina HiSeq platform on 450 *BRCA1/2* WT high-risk breast cancer patients. In a female patient <50 years of age, a novel frameshift mutation in *UBE2T*, c.415_418insAGCC, was detected and subsequently confirmed by amplicon resequencing on the MiSeq platform (>100 000× coverage). This 4-bp insertion appeared to be the consequence of a tetranucleotide repeat expansion (TAATAAGCCAGCCAGCCTTCC) and led to a frameshift at position c.416 in the open reading frame (UBE2T 415fs). The mutant reading frame encoded a protein of 158 amino acids total that included the first 136 WT amino acids normally present in UBE2T followed by 22 additional mutant amino acids (Supplementary Material, Fig. S7). In order to undisputedly demonstrate that this mutant UBE2T 415fs protein is not functional, we expressed the mutant open reading frame in *Ube2t−/−* DT40 cells. Western blotting confirmed the expression of the mutant UBE2T 415fs protein in the DT40 chicken cells (Fig. 5C). Challenging the stably transduced *Ube2t−/−* cells with increasing doses of MMC revealed that expression of the mutant UBE2T 415fs protein did not rescue the survival of transduced cells, in clear contrast to the *Ube2T−/−* DT40 cells that expressed either the UBE2T WT or 468fs protein (Fig. 5D).

## Discussion

The results of this study demonstrate that biallelic loss of function mutations in the *UBE2T* gene result in a new subtype of FA with features typical for ‘early’ FA genes. This is consistent with the UBE2T protein functioning as the main E2 conjugase for the FA pathway. The fact that loss-of-function germline mutations in both the ubiquitin E2 conjugase, *UBE2T*, and the E3 ligase, *FANCL*, result in a similar clinical and cellular phenotype is interesting, as UBE2T is not a member of the FA core complex, but is recruited independently to chromatin (28,29). On the other hand, UBE2T directly interacts with FANCL and seems to be the E2 conjugase in the cells that is absolutely necessary for the post-translational activation of FANCD2 and FANCI by monoubiquitination. Thus, the identification of *UBE2T* as the 18th FA gene is consistent with current models of the FA pathway and confirms again the intimate connection between the ‘early’ proteins in the FA pathway, including FANCA/B/C/E/F/G/L/M and UBE2T (T), which together mediate the central step activation step, monoubiquitination of FANCD2 and FANCI.

Despite the presence of FA-typical congenital abnormalities at birth in the patient, hematopoietic mosaicism resulted in normal peripheral blood values and normal bone marrow cellularity for the first 16 years of his life. Usually, somatic alterations in non-transformed cells remain beyond the detection limit, as the cells do not gain a distinct phenotype. However, in FA patients, there is a strong *in vivo* selective growth advantage for phenotypically normal, albeit genetically heterozygous, hematopoietic cells carrying a gain-of-function alteration resulting in a WT allele. Therefore, this rare reversion in a hematopoietic stem cell is dramatically amplified by cell differentiation and expansion over time and, therefore, can readily be studied in the progeny of the reverted stem cells in patients with mosaicism (17–19). As such, it is impossible to evaluate in this individual whether patients with biallelic *UBE2T* mutation will develop bone marrow failure. For this, the identification of FA-T patients without mosaicism might be informative.

The fact that the patient never experienced low platelet or leukocyte counts and, considering his thalassemia minor, had stable hemoglobin values already after the perinatal period (Supplementary Material, Fig. S1) strongly suggests that the aluY-mediated reversion of the maternally inherited exons 2–6 duplication in stem cells had already occurred early in life. As a reverted hematopoietic stem cell needs ample time to repopulate both the stem cell pool and the hematopoietic system, it appears likely that this monoallelic recombination event in the maternal allele occurred *in utero.* At the time of birth, the diagnosis of FA was based on the typical congenital abnormalities and two positive chromosomal breakage tests after exposure of PHA-stimulated peripheral blood T cells to DEB. Notably, however, the two chromosomal breakage tests performed during the first 2 weeks of life had already revealed that 15–20% of metaphases already did not have breaks despite the exposure to DEB. We therefore consider it possible that a low percentage of normal T cells without hypersensitivity to DEB was the first sign of ongoing reversion in the hematopoietic system. Difficulties in detecting the maternal allele with duplication of exons 2–6 in the patient's cDNA and genomic DNA from peripheral blood taken at 14 years of age (data not shown) also supports (nearly) complete repopulation.

The molecular diagnosis of FA was delayed in our patient due to the unique composition of a deletion and duplication along identical aluY elements within the locus of *UBE2T*. Remarkably, not a single base was clearly mutated in our patient compared with the *UBE2T* genomic sequence from the GRCh38.p2 genome assembly by Sanger or whole exome sequencing on either the maternal or the paternal *UBE2T* allele. Instead, only an abnormal distribution of exons and introns was present between the two alleles. In our case, even knowing the *UBE2T* gene defects, we could not find any reads in our whole exome sequencing data that spanned either the deletion or duplication and passed the quality filters, owing to the long polyT stretches within the aluY repeats that cannot be passed by current sequencing techniques. Therefore, although next-generation sequencing approaches are increasingly introduced into standard clinical care (53,54), it remains to be determined what the real detection rate of biallelic mutations in FA and other bone marrow failure patients will be in the CLIA-approved laboratory setting. Nevertheless, other techniques such as retroviral complementation or protein analysis of known FA genes are well established and can reliably be used to identify the unknown genetic defect, at least in a research setting (37,55–58).

Alu-mediated deletions have been identified in other genetic disorders (59,60) and also in other DNA repair/FA genes such as *BRCA2* and *FANCA* (61–64). These homology-mediated rearrangements/deletions are increasingly recognized as an important mechanism for introducing variations and causing mutations in the human genome (59,65). Mechanistically, these rearrangements are thought to be predominantly caused by non-homologous end joining repair, as HR usually requires a few hundred base pairs of homology and is restricted to the S phase of the cell cycle (66). While it is obvious that the reversion back to a normal allele in our patient's hematopoietic system was the result of an intragenic monoallelic recombination event that probably used two identical aluY elements as the matrix, it remains unclear which DNA repair pathways were ultimately used for the three different rearrangements within the *UBE2T* gene locus in this family. In order to systematically investigate possible events that could influence/trigger the genomic rearrangement between aluY repeats, we are currently generating multicolor fluorescent reporter constructs that reflect the structure of the maternal *UBE2T* allele with three identical aluY repeats in the same orientation. After stable integration of one copy into the genome of cells, we will inflict DNA double-strand breaks in these cells using I-*Sce*I sites and then analyze how *UBE2T* disruption influences the types and frequency of recombination events in these cells. We hypothesized that such genomic rearrangements are rather uncommon; however, the detection of these rare events was made possible in the patient's hematopoietic system by the strong *in vivo* survival advantage of the spontaneously corrected stem cell(s) and their progeny during the first 16 years of the patient's life.

Within this work, we could not elucidate whether the deletion and duplication of exons 2–6 deletion in *UBE2T* are founder mutations in humans due to their very low incidence: only two deletions in almost 1700 individuals. Strikingly, the duplication of exons 2–6 in *UBE2T* was not found outside of patient's family. However, difficulties in obtaining permission from the relatives of the patient's mother did not allow us to trace the origin of the duplication further.

Finally, the detection of a germline mutation in 1 of 450 high-risk breast cancer patients with normal *BRCA1/2* suggests that *UBE2T* also could be a very rare cancer susceptibility gene. The association of ‘early’ FA genes with FANCD2 monoubiquitination defects with increased cancer susceptibility is much weaker (67–69) compared with the ‘late’ FA genes such as *BRCA1*, *BRCA2*, *BRIP1*, *PALB2* and *RAD51C* (4,33–35). In addition, although aluY-mediated rearrangements in genes have been implicated in human disorders and also cancers (59–64), we did not find any rearrangement in the *UBE2T* locus in more than 800 breast/ovarian cancer patients from the Düsseldorf breast cancer clinic. Therefore, the question of whether loss-of-function mutations in *UBE2T* as *a* bona fide FA gene are associated with cancer susceptibility can only be answered in future studies involving a multi-institutional approach with large numbers of patients.

## Materials and Methods

### Human subjects and cells

The patient and his family are descendants of Italian immigrants to the USA who came from Sicily. The clinical care of the patient for the previous 16 years occurred at the Children's Hospital of Michigan in Detroit and at the Cincinnati Children's Hospital in Cincinnati. The chromosomal breakage test for the laboratory diagnosis of FA was performed by Arleen Auerbach, International FA Registry (5), Rockefeller University, New York. Skin fibroblasts from the patient were sampled for genetic analysis at the age of 2 years and were kindly provided by Dr Richard Harris, Cincinnati Children's Hospital, Cincinnati, OH. *FANCL*-deficient primary fibroblasts were a kind gift of Dr Auerbach, Rockefeller University, NY (70). Primary *FANCG*- and *FANCQ/ERCC4*-deficient reference fibroblast cells were kindly provided by Dr Detlev Schindler, Department of Human Genetics, University of Würzburg, Germany (2,38). Peripheral blood or DNA of healthy individuals was obtained with their informed consent. All studies were approved by the local institutional review boards/ethics committees. The DNA from the high-risk breast cancer patients was deidentified clinical samples from a subset of U.S. states allowing such anonymization and devoid of unique BRCA sequence variants that might have made it possible to re-identify the patients on the basis of their genotypes.

Fanconi anemia cells were grown in 5% O_2_ as described (13,50). Adherent cells were grown in high glucose DMEM, and non-adherent cells were cultured in RPMI1640. Both media were supplemented with 10% fetal bovine serum (FBS), 1% glutamine and 1% penicillin/streptomycin (all from Gibco BRL/Invitrogen). DT40 cell medium was additionally supplemented as described (28,29).

For chromosomal breakage testing, primary fibroblasts from a healthy donor (NL1433889), the *FANCG−*/*−* reference patient, and the *UBE2T−/−* patient FA 100166/1 were cultured in high glucose DMEM containing 20% FBS, 1% penicillin/streptomycin and 1 mm sodium pyruvate (all from Gibco BRL). Cells were cultured in 0, 0.01 or 0.1 µg/ml DEB for 48 h, then washed, and replenished with fresh medium. Subsequently, cells were exposed to 200 µg/ml colcemid for 7–8 h, harvested, and fixed using standard protocols (22,36). Metaphase spreads were prepared, GtG-banded and analyzed under a brightfield microscope.

### Plasmids and retroviruses

The retroviral and lentiviral vector backbones used in this study were described previously (13,50,71,72). The human *UBE2T* cDNA was a kind gift of Dr Anindya Dutta, University of Virginia School of Medicine, Charlottesville, VA (27). The open reading frame was amplified with primers 5′ *Not*I and 3′ *Bam*H I to add the necessary restriction sites for cloning into the pS91-IRES-NEO vector. cDNAs for human *FANCL*, *FAAP20*, *FAAP24*, *FAAP100, UBE2T* and *UBE2W* were purchased from Geneart (Invitrogen). The SV40 large T cDNA was a kind gift of Dr Valerie Schumacher, Human Genetics, Düsseldorf, Germany. All vectors were controlled by capillary Sanger sequencing prior to generating replication incompetent retroviral supernatants. Retroviral vectors were stable packaged in PG13 cells as previously described (13,50). Lentiviral vectors were produced with the GalvTM envelope as previously described (72,73). All adherent cells were transduced in the presence of 7.5 µg/ml polybrene (Sigma-Aldrich), and all non-adherent cells were exposed to virus on the recombinant fibronectin fragment CH-296 (Takara Shuzo) as described previously (74).

### Flow cytometry for cell cycle analysis and survival

Cell cycle analysis was performed as described (13,50). Briefly, G418 (Geneticin, Gibco BRL)- or puromycin (Sigma-Aldrich)-resistant primary fibroblasts were incubated for 3 days with increasing concentrations of MMC and then harvested, fixed, stained with propidum iodine and analyzed for their cell cycle distribution on a FACSCalibur (BD Biosciences). DT40 cells were grown for 3 days in increasing concentrations of MMC and analyzed on the FACSCalibur using propidium iodine staining to discriminate between live and dead cells. Flow cytometric data were analyzed using the Cell Quest (BD Biosciences) or Modfit (Verity Software House, Topsham, ME, USA) software programs. Data are shown as mean ± standard error of the mean (SEM) or are from a representative experiment.

### Western blotting

For FANCD2 western blotting, cell pellets were lysed and protein lysate was measured using the Pierce BCA Protein Assay Kit (Thermo Scientific, 23227). Briefly, 20 µg protein lysates were loaded onto a 7% tris acetate gel and run at 120 V for 7 h. The blot was transferred overnight onto nitrocellulose at 4°C in 20% methanol, Tris glycine buffer (1X) at 20 V. The blot was blocked in phosphate-buffered saline (PBS) with 0.01% Tween 20 (PBST) containing 5% skim milk for 30 min followed by FANCD2 antibody (Thermo Scientific, MA1-16570) diluted at 1:5000 for 3 h. Subsequently, the blot was washed three times in PBST and incubated with anti-mouse IgG horseradish peroxidase (HRP)-conjugated secondary antibody (Promega, W402B; 1:5000). Finally, the blot was washed three times, treated with the Pierce ECL Western Substrate, and exposed using a Bio-Rad imager. Analysis was performed with Bio-Rad Image Lab software 2.0. The blot was stripped using GM Biosciences One-Minute Western Blot Stripping Buffer (GM 6001), and the western protocol was repeated using RAD50 (GeneTex, GTX70228; 1:5000) as the primary antibody.

For the UBE2T western blotting, 20 µg protein lysates were run on a 4–12% Bis–Tris gel at 120 V for 1.5 h. The Western protocol was the same as above using the non-commercial UBE2T antibody that we have previously reported (44) (1:1000) and anti-sheep as the secondary antibody (Promega). The blot was stripped and incubated with antibody to glyceraldehyde-3-phosphate dehydrogenase (GAPDH; GeneTex, GTX627408; 1:5000) or β-actin (Sigma-Aldrich, A5441; 1:5000).

To detect FANCI, 40 µg protein lysate was separated on an 8% Tris–glycine gel at 80 V for 0.5 h plus 180 V for 2 h. The blot was transferred 2 h onto nitrocellulose in 20% methanol, Tris glycine buffer (1X) at 30 V. The blot was blocked in Tris-buffered saline (TBS) with 0.1% Tween 20 (TBST) containing 5% skim milk for 30 min followed by FANCI antibody (Bethyl, A300-212A) diluted at 1:2000 for 3 h. Subsequently, the blot was washed three times in TBST and incubated with anti-rabbit IgG horseradish peroxidase (HRP)-conjugated secondary antibody (Promega).

### Whole exome sequencing (WES)

WES on the primary FA 100166/1 cells was performed at the Center for Inherited Disease Research (CIDR, Johns Hopkins University) as described previously (75). Briefly, exonic sequences were captured using the Agilent SureSelect 51 Mb Human All Exon Kit, and paired-end sequencing was performed on the Illumina HiSeq 2000 system, using Flowcell version 3 and TruSeq Cluster Kit version 3. Primary analysis was done using HiSeq Controls Software and Runtime Analysis Software. The CIDRSeqSuite version 3.0.1 pipeline was used for secondary bioinformatics analysis, which consists mainly of alignment using Burrows Wheeler Aligner version 0.5.9 to the human genome reference sequence (build hg19) and applying the Genome Analysis Toolkit (GATK) version 1.4-29-gcd352f5 to perform local realignment and base quality score recalibration. Duplicate molecules were flagged and mate-pair information synchronized using Picard version 1.57, and the GATK Unified Genotyper was used for variant calling. ANNOVAR was used to annotate variants for location and predicted effect on the protein, corresponding gene information, allele frequencies across multiple databases, and predicted variant effects.

WES using the MySeq platform was performed on genomic DNA extracted by QIAsymphony using the DSP DNA Midi kit (Qiagen) from peripheral blood of 450 *BRCA1/2* wild-type breast cancer patients with an age at diagnosis <50 years, anonymized from the US clinical testing sample flow of Myriad Genetic Laboratories, using the Nextra Enrichment Kit (DC-121-1208, Illumina). JAligner was used for the initial mapping, followed by the variant calling, call quality assessment and review using in-house-developed software. The novel frameshift mutation in *UBE2T*, c.415_418insAGCC, was subsequently confirmed by amplicon resequencing on the MySeq platform (with the amplicon spanning chromosome 1 hg19/GRCh37 locations 202 302 051–202 302 350 using primer sequences GTTTCTGTCTTGCATGCTTCTC and CCTCTGCAACACATATCCTACC). To avoid alignment efficiency biases caused by the presence of the insertion, the allele ratios were subsequently requantified using local realignment with the reference and mutated sequences.

### Sanger sequencing and PCR of cDNA

Screening for mutations in *UBE2T* was carried out using direct genomic sequencing. The PCR primers designed to amplify and sequence all exons and adjacent introns of the *UBE2T* gene (RefSeq: NM_014176.3) are listed in Table 1. Sequencing was performed using an ABI 3730xl DNA analyzer (Applied Biosystems). To detect the deletion of exons 2–6 in *UBE2T*, RNA isolated from peripheral blood, T cells expanded on CD3/CD28 immobilized antibodies (55), or fibroblasts using the RNeasy Mini Kit (Qiagen) was retrotranscribed with RNase H+ MMLV reverse transcriptase (iScript™ cDNA Synthesis Kit, Bio-Rad) and PCR-amplified with exon primers 1F and 7R. To detect the duplication of exons 2–6 in *UBE2T*, forward primer 6F (exon 6) in combination with any reverse primer located in exons 1–7 as well as with forward (2F) and reverse (2R) primers located in exon 2 were used (EmeraldAmp MAX PCR Master Mix [Takara] and/or Platinum^®^Taq DNA Polymerase [Invitrogen]). All abnormally sized PCR fragments corresponding to the mutant allele(s) were gel-extracted (Gel extraction kit, Qiagen) and cloned into pGEM-T vector (pGEM^®^-T Easy Vector System, Promega). Positive colonies were purified using the Wizard^®^ Plus Minipreps DNA Purification System (Promega) and capillary sequenced. Long-range PCR was performed to detect and further characterize duplicated and deleted alleles using genomic DNA. The same PCR conditions were used.

### PCR for detection of the duplication and deletion of exons 2–6 in genomic DNA

Genomic DNA isolated from whole blood, fibroblasts or a newly established EBV-transformed B-cell line using QiaAmp DNA Mini Kit (Qiagen) were PCR-amplified with forward primer 1_7F, reverse primer 1_7R and additional reverse primer ivs1R (Fig. 2C, Table 2) using EmeraldAmp MAX PCR Master Mix and/or Platinum^®^Taq DNA polymerase. A band of 1750 bp was amplified from the deleted allele with the primers 1_7F and 1_7R, whereas a second reverse primer ivs1R allowed amplification of the control band of 1066 bp (Fig. 2C). For detection of the duplication, additional forward intron 5 (ivs5F) and reverse intron 1 (ivs1R) primers (Fig. 2C, Table 2) were designed in close proximity of repetitive elements. These two primers when combined with exon 7 (7R) primers allowed us to co-amplify a control band of 1360 bp and a duplication-specific band of 769 bp in the same reaction (Fig. 2C). All PCR fragments corresponding to the mutant alleles were gel-extracted (Gel extraction kit, Qiagen) and cloned into pGEM-T vector (pGEM^®^-T Easy Vector System, Promega). Positive colonies were purified using the Wizard^®^ Plus Minipreps DNA Purification System (Promega) and capillary sequenced.

**Table 2.** Primers

cDNA primers for cloning of *UBE2T* into S91-IRES-NEO	
5′ *Not*I	CATGCGGCCGCATGCAGAGAGCTTCA
3′ *Bam*H I	GTAGGATCCCTAAACATCAGGATG
cDNA primers	
1F	AGTCAGAGGTCGCGCAGGCGCTG
1R	CCCTCACAACGCAGCAA
2F	TGCATCCCAGGCAGCTCTTA
2R	TAGAAGGAACCACACACAGTTC
3R	CATAAGGTGTGTTGGCTCCACCTA
4R	CATCCAGACAAATCCTTCCAGCAG
5R	TGGGTTCTGACATGAGCAGCTGAA
6F	CAAGAATGCCAGACAGTGGACAGA
6R	CTTGAGGAAGGCTGGCTTATTA
7R	GGTAGGCAACTTAGATCACCTTGGCA
Genomic primers for the duplication^a^	
ivs5F	CCTCTGCAACACATATCCTACC
ivs1R	CCTCTGTGCGTCTACATCTATTT
e7R	TCTATGCCTACTAGCTGACTGG
Genomic primers for the deletion^a^	
1_7F	GCTTCTTTCCCGGTGGATTA
1_7R	CCCAGACACACATTCAGGATAAA
ivs1R	AAACTCATGCTTCAGCCACACTGC
Primers for NMD detection^a^	
Exon 1	tgtaaaacgacggccagtAGTCAGAGGTCG CGCAGGCGCTG^b^
duplication-specific	gcctgggatgcActtttgtttctg^c^

^a^All primers shown as 5′ to 3′, F = forward, R = reverse.

^b^Contains the M13 forward binding site (small letters).

^c^Underlined A indicates the site of the junction in cDNA.

For detecting the deletion and duplication of exons 2–6 in UBE2T in the genomic DNA of healthy donors from Germany and Italy and from German breast cancer patients, DNA was extracted from the blood samples using the DNeasy 96 Blood & Tissue Kit (Qiagen). For the fibroblasts and lymphoblastoid cell lines (LCLs), DNA was extracted using QIAShredder and QIAamp DNA Mini Kit (both Qiagen) and amplified by PCR with 30 cycles. PCR was performed on 50 ng template with 250 pmol of each primer for 30 cycles using the primers listed in Table 2. The deletion analysis was amplified by my-Budget Taq-DNA-polymerase (Biobudget) supplemented by 12.5 mm MgCl_2_ per reaction. The duplication reaction contained HotStarTaq DNA polymerase (Qiagen) and 2.5 mm MgCl_2_. The PCR products from the blood samples, normal LCLs as a negative control, the patient's fibroblasts as a positive control, and a water control were separated on 0.8% agarose gels, stained with ethidium bromide and visualized.

### Celltiter 96 AQueous non-radioactive cell proliferation assay (MTS assay)

Immortalized 3000 cells of 100166/1T cells transduced with the retroviral control or UBE2T vectors were seeded in wells of a 96-well plate. Five wells were plated for each dose of genotoxin. Cells were allowed to adhere for a minimum of 8 h before addition of the indicated doses of genotoxin. Cells were left to grow for 4 days at 37°C in a humidified, 5% CO_2_ atmosphere before MTS assays were performed according to the manufacturer's instructions (Promega). Briefly, 20 μl of combined MTS/PMS solution was added to cells in each well, and cells were incubated for 1.5 h at 37°C in a humidified, 5% CO_2_ atmosphere before the absorbance at 490 nm was measured using an enzyme-linked immunosorbent assay plate reader. Data are presented as the mean of three independent experiments.

### Psoralen-induced ICL recruitment assays

Cells were plated on glass-bottomed dishes (WPI) and pre-treated with 50 μM psoralen (Sigma) 1 h before psoralen was activated using a 360-nm laser scanning 5X at 25% energy across cell nuclei (PALM microscope, Carl Zeiss). Cells were fixed 30 min post-laser irradiation with 2% paraformaldehyde for 10 min at room temperature. Cells were then permeabilized with 0.2% Triton X-100 in PBS for 10 min at room temperature before several washes in PBS and incubation in blocking solution (PBS containing 3% IgG-free bovine serum albumin (Jackson Immunoresearch) and 0.2% Tween 20) for 1 h. Cells were then incubated with the indicated primary antibodies (1 μg/ml) in blocking solution for 1 h. After extensive washing in PBS containing 0.2% Tween 20, cells were incubated with secondary antibodies (2 μg/ml) conjugated to fluorescein isothiocyanate (FITC; for γ-H2AX) or Texas Red (for FANCD2) for 45 min. Cells were washed thoroughly and stained with DAPI-Hydromount for 5 min before being covered with a glass coverslip. Cell staining was viewed using a Deltavision DV3 widefield deconvolution microscope mounted on a Nikon Diaphot inverted microscope, and images were deconvolved after acquisition. The γ-H2AX antibody was purchased from Millipore (#05-636), the FANCD2 monoclonal antibody from Abcam (#ab2187), and the secondary Alexa Fluor-conjugated antibodies were obtained from Invitrogen.

### NMD in FA patient 100166/1 fibroblasts

One day prior to cycloheximide treatment, fibroblasts were plated in 6-well tissue-culture dishes such that the cells were 80–90% confluent the following day. The cells were treated with 25, 50 or 100 μg/ml cycloheximide dissolved in dimethyl sulfoxide (DMSO; Sigma-Aldrich). As additional controls, cells were treated with DMSO alone or just grown in medium without DMSO. After 6 h, the cells were washed twice with PBS and lysed directly in the well for isolation of total mRNA using the Qiagen RNeasy kit. cDNA was synthesized using random hexamers and the iScript cDNA Synthesis Kit according to the manufacturer's recommendations (Bio-Rad). PCR was performed with the EmeraldAmp MAX PCR Master Mix and gene-specific primers. The forward primer was located within exon 1 (Table 2) and contained the M13 forward binding site. The reverse primer was specific for the UBE2T duplication (Table 2) as it encompassed a unique region of the mutated cDNA that is part of the normally non-translated 5′ region of exon 2 and the 3′ region of exon 6 at the junction site. The PCR product was 565 bp.

## Supplementary Material

Supplementary Material is available at *HMG* online.

## Funding

Research in our laboratories was supported by NIH R01 CA155294-01 and a BMBF grant ‘Inherited bone marrow failure syndromes’ (to H.H.) and by the Scottish Institute for Cell Signalling by the Wellcome Trust strategic award grant 097945/B/11/Z (to A.A.). Helmut Hanenberg is supported by the Lilly Foundation Physician/Scientist initiative and the Walther Cancer Foundation. Indiana University is a Center for Excellence in Molecular Hematology (P30). Funding to pay the Open Access publication charges for this article was provided by the COAF fund from the Wellcome Trust to the University of Dundee.

## Supplementary Material

Supplementary Data
