# Supplementary material for: AluY-mediated germline deletion, duplication and somatic stem cell reversion in UBE2T defines a new subtype of Fanconi anemia
Source: Hum Mol Genet. 2015 Jun 17;24(18):5093–108. doi: 10.1093/hmg/ddv227 (PMC4550815; doi:10.1093/hmg/ddv227)
Supplement: Supplementary Data [file supp_24_18_5093__index.html]

AluY-mediated Germline Deletion, Duplication and Somatic Stem Cell Reversion in UBE2T Defines a New Subtype of Fanconi Anemia — AluY-mediated germline deletion, duplication and somatic stem cell reversion in UBE2T defines a new subtype of Fanconi anemia — AluY-mediated germline deletion, duplication and somatic stem cell reversion in UBE2T defines a new subtype of Fanconi anemia — Supplementary Data 

# AluY-mediated germline deletion, duplication and somatic stem cell reversion in *UBE2T* defines a new subtype of Fanconi anemia

## Supplementary Data

Supplementary Data

- Supplementary Data - Docx file
