# Supplementary material for: AluY-mediated germline deletion, duplication and somatic stem cell reversion in UBE2T defines a new subtype of Fanconi anemia
Source: Hum Mol Genet. 2015 Jun 17;24(18):5093–108. doi: 10.1093/hmg/ddv227 (PMC4550815; doi:10.1093/hmg/ddv227)
Supplement: Supplementary Data [file supp_ddv227_ddv227supp.docx]

**SUPPLEMENTARY FIGURE LEGENDS**

**
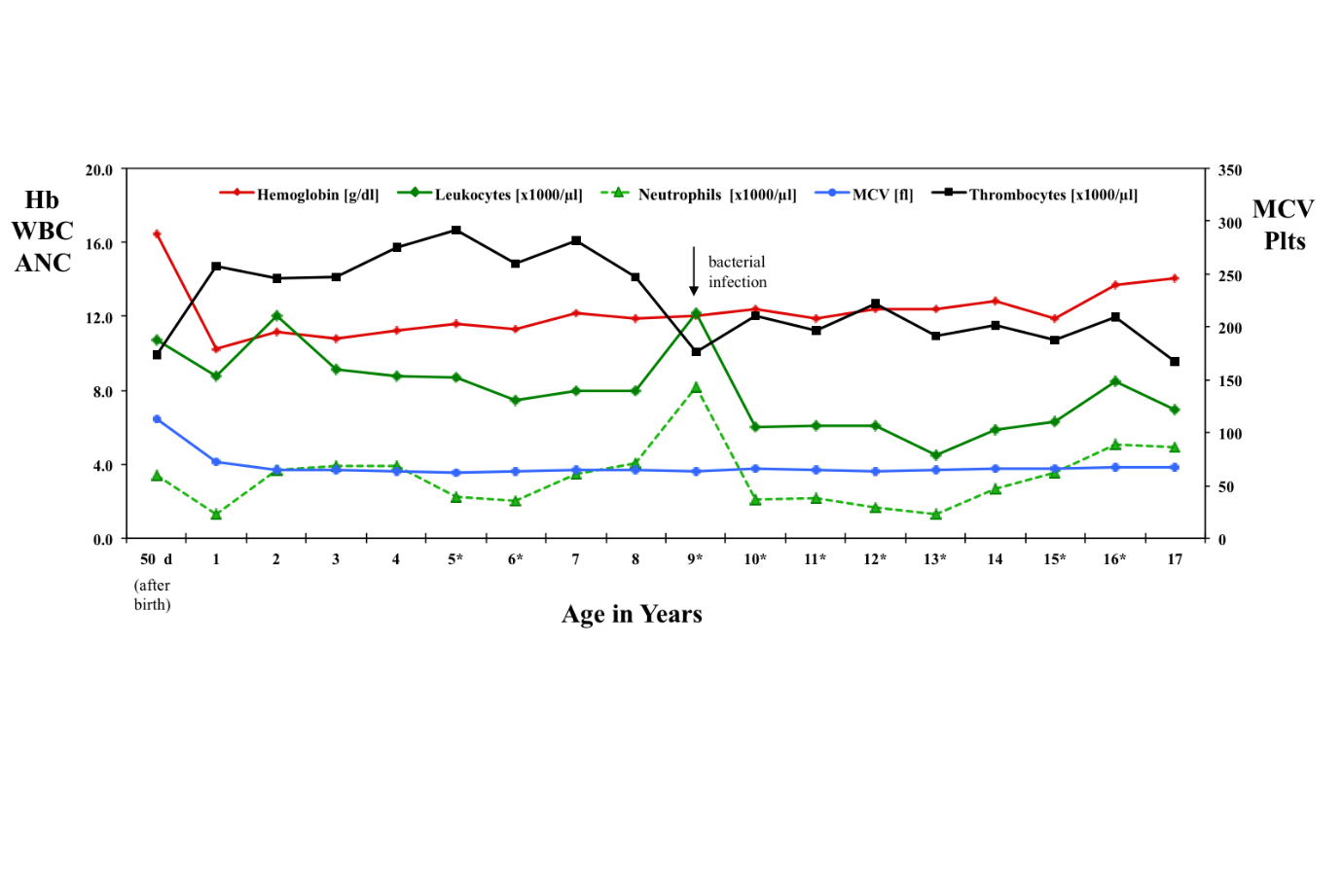
**

**Figure S1: Time course of peripheral blood values since birth.** Hemoglobin (Hb), leukocyte count (WBC), absolute neutrophil count (ANC), mean corpuscular volume (MCV), and thrombocyte count (Plts) are shown as average values for the first 50 days after birth and then as averages over 12 months in each of the next 16 years. In years marked with an asterix (*), only counts from a single time point were available.

**
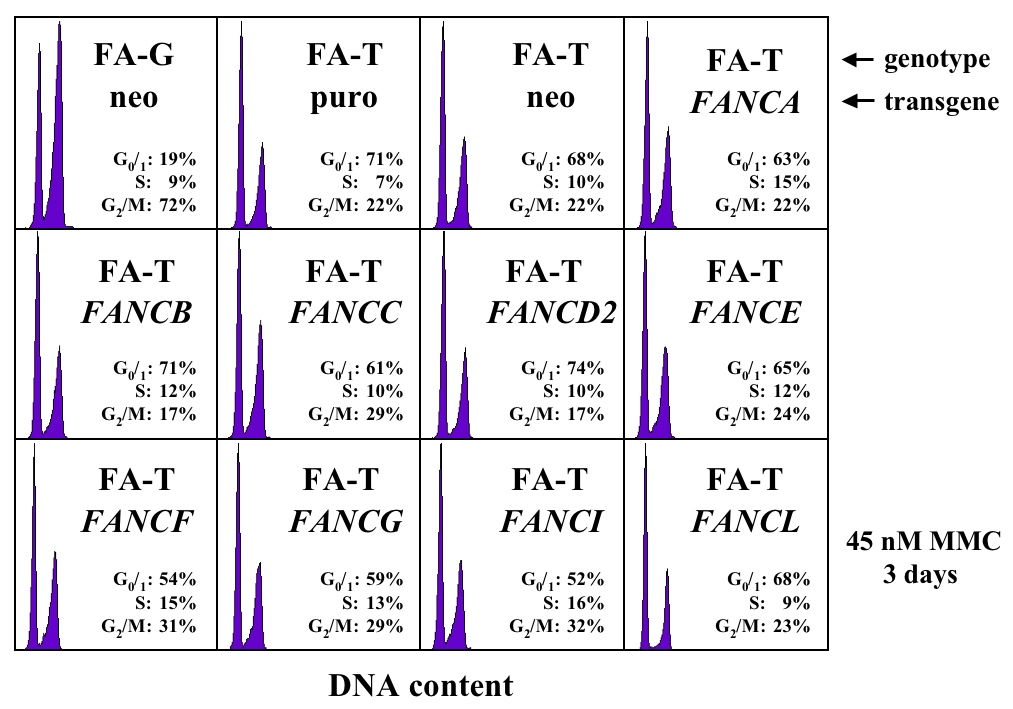
**

**Figure S2: Flow cytometric cell cycle analysis of primary fibroblasts transduced with ‘early’ FA genes grown after 3 days in 45 nM MMC.** FA-G: primary *FANCG-/-* fibroblasts (70) transduced with the control vector (neo). FA-T: primary patient 100166/1 fibroblasts transduced with the control vectors (neo or puro) or the *FANCA/B/C/D2/E/F/G/I/L* coexpressing vectors. The distribution of cells in G_0_/G_1_, S, and G_2_/M arrest from a representative experiment is shown as analyzed by Modfit.

**
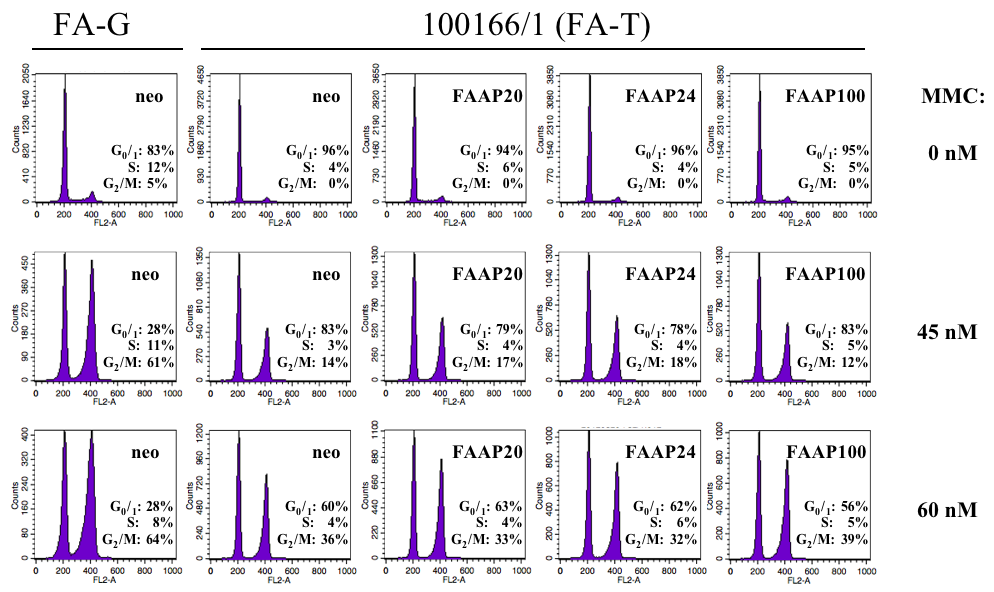
**

**Figure S3: Flow cytometric cell cycle analysis of primary fibroblasts transduced with the FA candidate genes *FAAP20, FAAP24* and *FAAP100* after 3 days in 0, 45 and 60 nM MMC.** FA-G: primary *FANCG-/-* fibroblasts (70) transduced with the control vector (neo). FA-T: primary 100166/1 fibroblasts transduced with the control vector (neo) or the FAAP20-, FAAP24- and FAAP100-coexpressing vectors. The distribution of cells in G_0_/G_1_, S, and G_2_/M arrest from a representative experiment is shown as analyzed by Modfit.

**
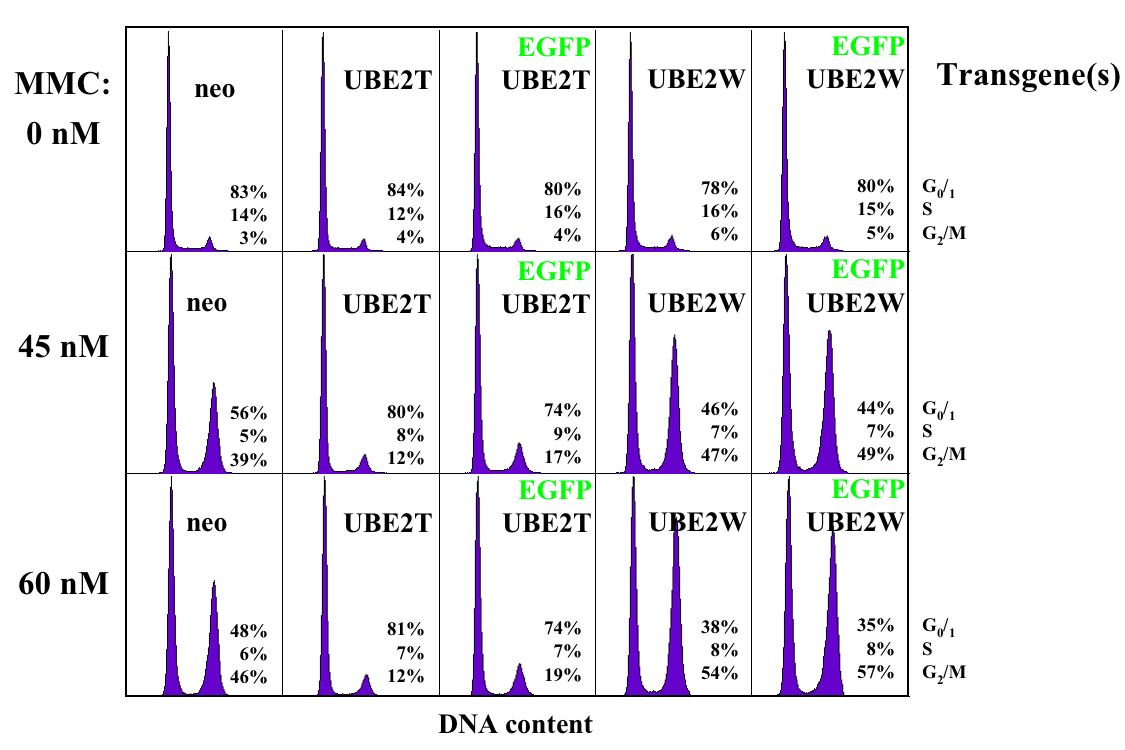
**

**Figure S4: Flow cytometric cell cycle analysis of primary FA 100166/1 fibroblasts transduced with the cDNAs for the two E2 conjugases *UBE2T* and *UBE2W* and coexpressed with the neomycin resistance gene, *npt II*, via an IRES element either as a 5’EGFP fusion construct or alone.** Cells were grown for 3 days in 0, 45 or 60 nM MMC and then analyzed on a FACSCalibur (BD Bioscience). The distribution of cells in G_0_/G_1_, S, and G_2_/M arrest from a representative experiment is shown as analyzed by Modfit.

**
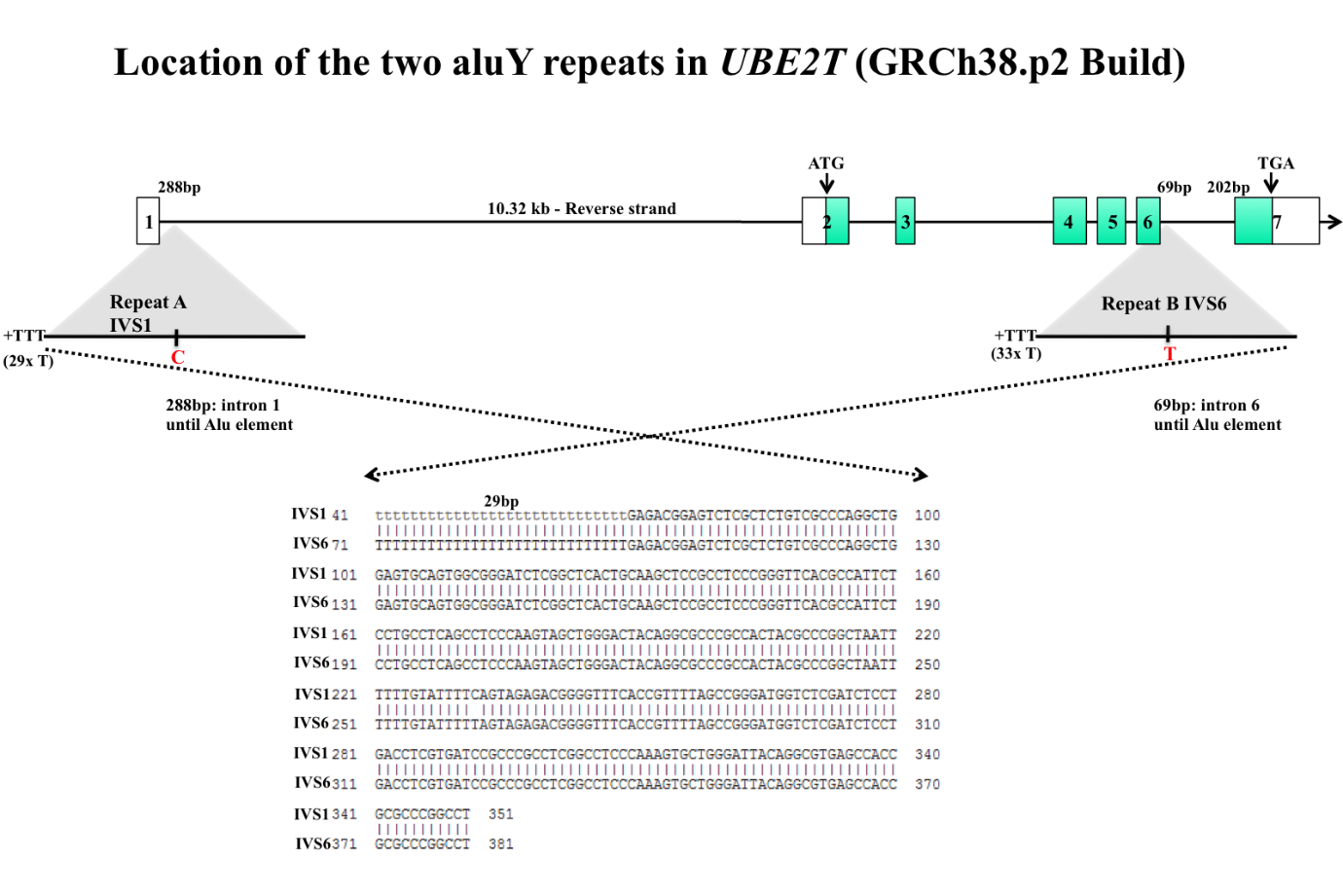
**

**Figure S5: Location of the two AluY Repeats in *UBE2T* (GRCh38.p2).** The aluY repeats are located as shown in introns 1 and 6 and start 288 and 69 bp within the introns, respectively. In the GRCh38.p2 build, both aluY elements are identical except a C/T mismatch at position 192. In addition, the aluY element in intron 6 has four additional Ts at the 5’ end of the repeat.


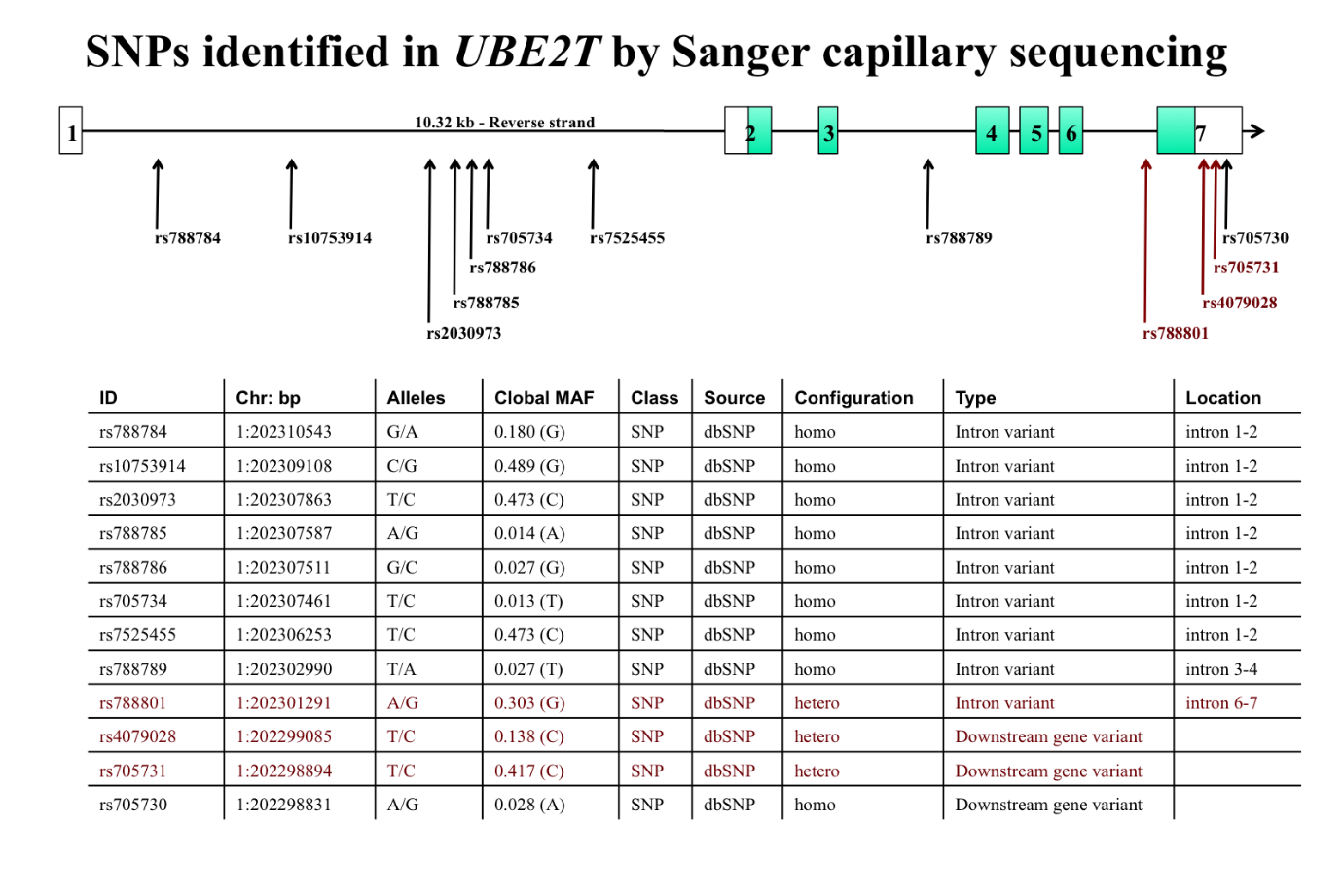


**Figure S6: SNPs identified in *UBE2T* by Sanger capillary sequencing.** All SNPs except for rs788789, rs4079028, and rs705731 (marked in red) were found in the homozygous state.


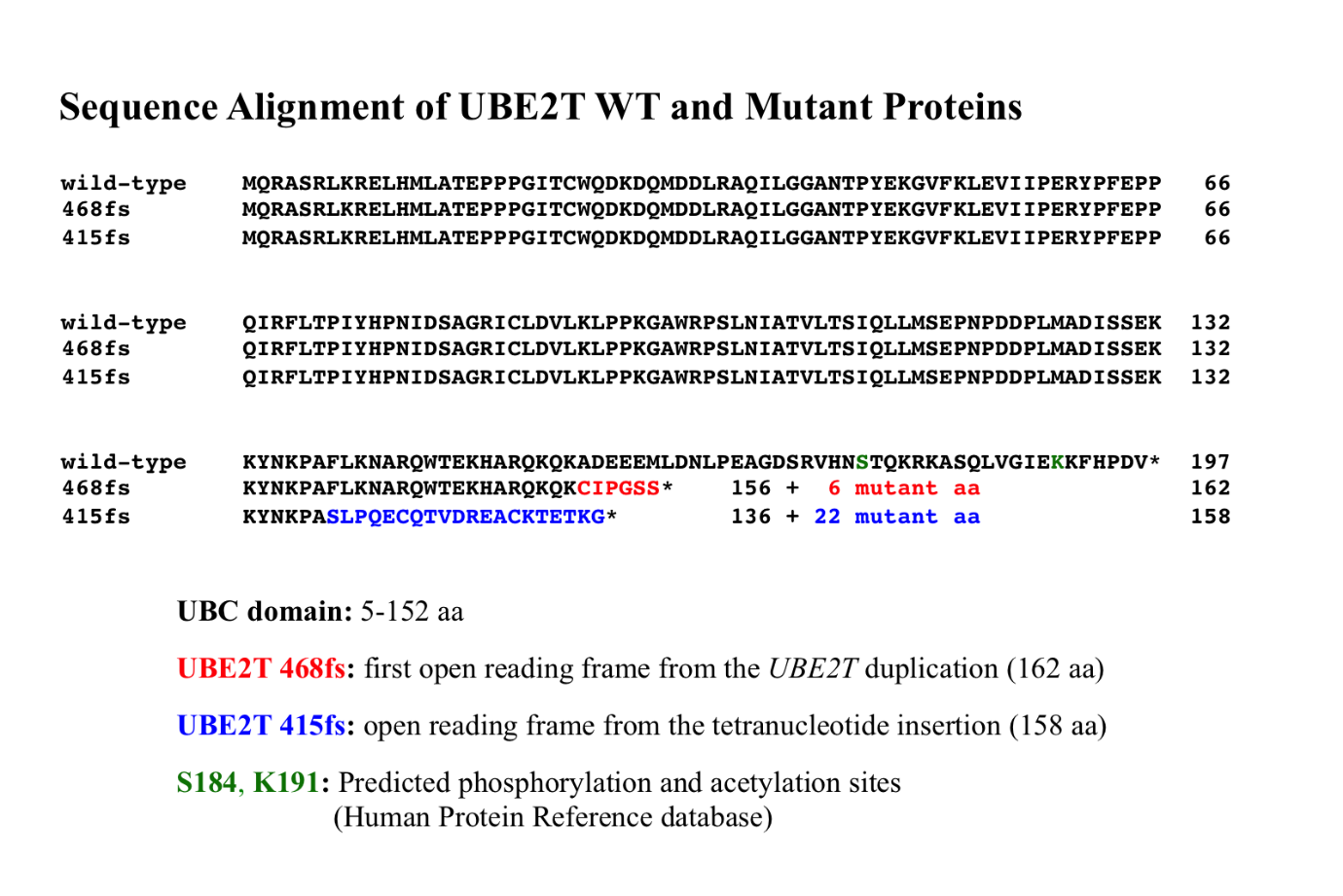


**Figure S7: Sequence alignments of WT UBE2T and mutant proteins.** The maternal *UBE2T* allele with the duplication of exons 2–6 encodes a shorter UBE2T protein (UBE2T 468fs) of 162 aa, which contains a complete UBC domain (5–152 aa). The mutant UBE2T allele with the tetranucleotide insertion encodes a mutant protein of 158 aa that does not contain the complete UBC domain. Mutant amino acids in UBE2T 468fs (red) and 415fs (blue) are shown.
